# Urine Protein/Creatinine Ratios during Labor: A Prospective Observational Study

**DOI:** 10.1371/journal.pone.0160453

**Published:** 2016-08-01

**Authors:** Vaya W. Tanamai, Brandon-Luke L. Seagle, Judy Y. Yeh, Bethany Brady, Corrie B. Miller, Salvador Sena, Jessica Dodge, Shohreh Shahabi, Robert Samuelson, Errol R. Norwitz, Guoyang Luo

**Affiliations:** 1 Department of Obstetrics, Gynecology and Reproductive Sciences, Western Connecticut Health Network, Danbury, Connecticut, United States of America; 2 Department of Pathology and Laboratory Medicine, Western Connecticut Health Network, Danbury, Connecticut, United States of America; 3 Department of Obstetrics and Gynecology, Prentice Women’s Hospital, Northwestern University, Feinberg School of Medicine, Chicago, Illinois, United States of America; 4 Department of Obstetrics & Gynecology, Tufts Medical Center, Boston, Massachusetts, United States of America; Emory University, UNITED STATES

## Abstract

**Purpose:**

To evaluate the utility of urine protein/creatinine ratio (uPCR) measurements among healthy parturients at term we performed a prospective cohort study at a community teaching hospital.

**Methods:**

Serial urine samples were collected. Ninety-three women contributed 284 urine samples. uPCRs were determined. Multiple imputation and paired sampled analysis was performed when appropriate.

**Results:**

Two-thirds (63/93) of women had at least one measured uPCR ≥ 0.3. One-third (31/93) had a uPCR ≥ 0.3 at admission, including 39.1% (9/23) of women not in labor. Median (IQR) uPCRs increased during labor and after delivery: latent phase/no labor, 0.15 (0.06–0.32); active phase, 0.29 (0.10–0.58); early postpartum, 0.45 (0.18–1.36) (all p < 0.04). Median uPCRs were significantly < 0.3 in the latent phase and significantly > 0.3 in the immediate postpartum period (p < 0.01). Women who labored before cesarean delivery had the highest early postpartum uPCRs: median (IQR) 1.16 (0.39–1.80). A negative urine dipstick protein result did not exclude uPCR ≥ 0.3. uPCRs were similar when compared by method of urine collection.

**Conclusion:**

uPCR ≥ 0.3 is common among healthy women with uncomplicated pregnancies at term. uPCR increases during labor and is not a reliable measure of pathologic proteinuria at term or during the peripartum period.

## Introduction

Proteinuria is an important criterion in diagnosing preeclampsia in pregnancy [1]. A 24 hour urine collection remains the “gold standard” for the estimation of proteinuria during pregnancy. It is performed commonly at term to distinguish nonproteinuric gestational hypertension from preeclampsia. The cut-off value for pathologic proteinuria in pregnancy (accepted as ≥ 300 mg of total protein per 24 hours) was established using samples from pregnancies without preexisting medical conditions and prior to the onset of labor [2,3]. A 24 hour urine collection is time-consuming to collect and is difficult to collect during labor. For these reasons, attention has recently turned to the urine protein/creatinine ratio (uPCR) as a convenient alternative. The 2013 ACOG Task Force on Hypertension in Pregnancy included a random uPCR ≥ 0.3 as one of the diagnostic criteria for preeclampsia [1]. However, how the uPCR ≥ 0.3 threshold performs at term with or without labor has not been systematically examined.

Initial studies correlating single-void uPCR measurements and 24 hour total urinary protein estimations studied medically complicated pregnancies prior to the onset of labor [4]. These and other studies suggest that a uPCR < 0.15 is helpful to exclude significant proteinuria (accepted as ≥ 300 mg/24 h), but that mid-range uPCR cut-offs (such as 0.3) have a poor sensitivity and specificity for making this distinction [4,5]. There is also evidence to suggest that uPCR measurements vary with hydration status. In one recent publication, De Silva et al. reported that 39% of 233 samples collected from 160 women with high-risk pregnancies between 20–32 weeks of gestation had a single uPCR ≥ 0.3 [6]. In their study, uPCR was ≥ 0.3 among 94% of women with dilute urine (defined as a urine creatinine concentration < 34 mg/dL) as compared with 16.4% among women with non-dilute urine (p < 0.001) [6]. Despite these data, a single-void uPCR < 0.3 is now widely used to rule out preeclampsia without severe features among women presenting with hypertension at term.

The last report we could locate on intrapartum proteinuria was published in 1956 and studied medically complicated high-risk pregnancies using an outdated methodology [7]. To measure proteinuria at term and during labor by uPCRs and to evaluate the utility of the uPCR ≥ 0.3 criterion in this setting, we performed a prospective observational study of uPCRs among healthy women with low-risk pregnancies admitted for delivery at term.

## Materials and Methods

A prospective observational cohort study of proteinuria during labor was carried out at Danbury Hospital in Danbury, CT between May 12, 2014 and August 26, 2014. Low-risk women were invited to provide urine samples for protein estimation before, during, and after delivery. Women were identified on admission to Labor & Delivery and monitored from hospital admission to discharge. Inclusion criteria included a live single intrauterine pregnancy at term (gestational age ≥ 37.0 weeks) and admission for a planned delivery (either spontaneous labor, induction of labor, or scheduled cesarean). Exclusion criteria included maternal age < 18 years; preexisting hypertension (defined as a systolic BP ≥ 140 mmHg and/or diastolic BP ≥ 90 mmHg documented on at least one occasion in the prenatal record); hypertension documented for the first time on admission (defined either as a persistent increase in systolic BP ≥ 140 mmHg and/or diastolic BP ≥ 90 mmHg for more than 15 minutes or a single severe BP measurement of systolic BP ≥ 160 mmHg and/or diastolic BP ≥ 110 mmHg); ≥ 1+ proteinuria by urine dipstick on admission; recent bed rest (defined as ≥ 2 weeks of modified bed rest); ≥ 6 cm cervical dilation before collection of the first urine specimen; current urinary tract infection (UTI) or a history of recurrent UTI; any underlying chronic medical condition (such as renal disease, cardiovascular disease, autoimmune diseases, pre-gestational diabetes, and treatment-requiring gestational diabetes); and medications that could have affected renal function. Only healthy women with uncomplicated pregnancies and without comorbidities that might affect renal functions were enrolled. Women with ≥ 1+ proteinuria on urine dipstick at admission were excluded because of concerns of an underlying unrecognized renal disorder that may have introduced bias into the study. A diagnosis of UTI was excluded if a woman had no characteristic symptoms, no recent diagnosis of UTI at the time of admission, and no evidence of UTI on routine dipstick. Twin gestations were also excluded as levels of proteinuria are known to be increased in multiple gestations [8,9]. The study was approved by the Institutional Review Board of the Biomedical Research Alliance of New York (protocol number 14-02-113-337). Each woman provided written informed consent. Recruitment was stopped after consenting 100 women, when data analysis clearly showed that elevated uPCRs were common among the cohort.

Urine specimens were collected by midstream clean catch if membranes were intact and there was no evidence of vaginal bleeding. Otherwise, specimens were collected by sterile catheterization. Urine specimens were collected at the following time points: (a) on admission to the hospital (latent phase labor or not in labor); (b) during active labor (regular phasic uterine contractions and cervical dilation ≥ 6 cm); (c) in the immediate postpartum (recovery) period (within 6 h of delivery); and (d) in the later postpartum period (approximately 12–24 h postpartum). Participants were allowed to decline donation of any specimen. For each specimen, we recorded the collection time, method of collection (catheterization or clean catch), and urine dipstick protein measurement. Urine dipsticks were performed using Chemstrip^®^ 7 test strips (Roche Diagnostics, Indianapolis, IN). Urine specimens were maintained at room temperature and transported immediately to the hospital clinical laboratory for analysis. Quantitative assessments of urine protein and creatinine concentrations were performed using the VITROS^®^ 5.1 FS Chemistry System (Ortho Clinical Diagnostics, Rochester, NY) and data expressed in mg/dL [10]. Both tests are dry slide assays that utilize ultraviolet-visible reflectance spectrophotometry. The VITROS^®^ protein assay uses a molybdenum (VI) pyrocatechol violet dye-binding methodology to generate a colored protein-dye complex that is measured at 670 nm. As with most dye-binding assays for urine protein measurement, the VITROS^®^ method preferentially detects albumin over globulins and is calibrated using albumin standards. The VITROS^®^ creatinine assay uses a sequential enzymatic methodology that involves stepwise conversion of creatinine to creatine, sarcosine, and then to glycine, formaldehyde, and hydrogen peroxide via the enzymes creatinine amidohydrolase, creatine amidohydrolase, and sarcosine oxidase, respectively. The hydrogen peroxide that is produced then oxidizes a leuco dye in the presence of peroxidase to generate a color that is measured at 670 nm.

Maternal and infant demographic and clinical data were abstracted from the electronic medical record. Data analysis, statistical testing, and figure creation was performed using the R statistical programming language [11]. Appropriate statistical tests were used to compare maternal demographic and clinical outcome data. The specific statistical test used for each comparison is listed with the Results for convenient reference. Urine protein concentrations below the threshold of detection (< 5 mg/dL) were included in all analyses as 0 mg/dL to prevent biasing the results toward higher uPCRs, especially in dilute samples (i.e., those with low creatinine concentrations). For paired-sample comparisons of repeated uPCR measurements from the same women, samples collected from 92 women who donated samples at admission, during active labor, or during the early postpartum period (< 6 hours after delivery) were used; later postpartum samples (> 6 hours from delivery) were not used for paired-sample analyses. The number of samples missing included 2/92 at admission, 26/92 for active labor, and 23/92 for early postpartum, for an overall data completeness of 81.5% (225/276). Multiple imputation data sets (m = 5) were created and used for paired-sample comparisons with missing data points [12]. Paired-sample comparisons of uPCRs were performed using the Wilcoxon signed-rank test.

Where indicated with Results, uPCRs measurements were compared as independent samples when grouped by method of sample collection, urine dipstick protein measurement, or when compared to the uPCR = 0.3 criterion using the Mann-Whitney u test for two groups (when cases were grouped by method of sample collection or against the uPCR = 0.3 threshold) or Kruskal-Wallis test for more than two groups (when grouped by urine dipstick protein measurement).

## Results

### Patient and Sample Characteristics

**Fig 1:**
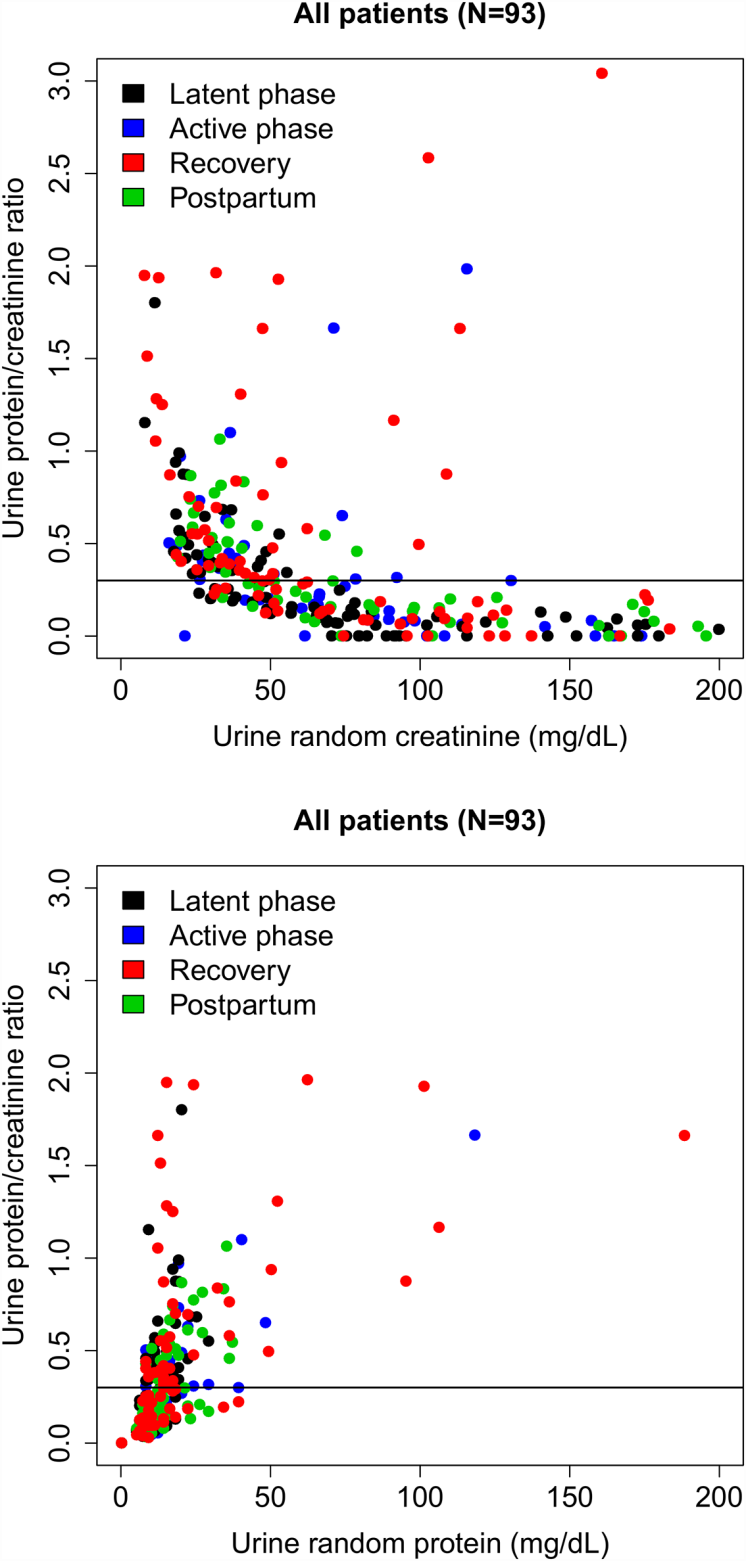
Urine protein/creatinine ratio (uPCR) by urine creatinine and protein concentrations. uPCR measurements plotted by urine random creatinine (top) and protein (bottom) concentrations and colored by phase of labor and delivery admission. Horizontal line on each graph represents uPCR = 0.3.

During the study period, 371 women were screened for eligibility. Of the 156 women who were eligible, 69% (100/156) consented to participate. Seven women progressed in labor to ≥ 6 cm cervical dilation before the first urine specimen could be collected and were excluded. A total of 284 urine specimens were collected from 93 women. The demographic and clinical data for the 93 study participants are shown in Table 1. Of these women, 53 (57.0%) delivered vaginally, 17 (18.3%) had cesarean deliveries after labor, and 23 (24.7%) delivered by scheduled cesarean before the onset of labor. Ninety specimens were collected before active labor, 66 during active labor; 69 within 6 h of delivery; and 59 after 6 h from the delivery time. Eleven women donated one specimen; 13 women donated two specimens; 37 women donated three specimens; 24 women donated four specimens; and eight women donated five specimens. Median and mean uPCRs were 0.23 and 0.46, respectively, with a range of 0 to 8.87. The 95^th^ percentile uPCR was 1.64, and 43% (122/284) of all measured uPCRs were ≥ 0.3. Proteinuria was undetectable in 14% (40/284) of samples. Fig 1 demonstrates the uPCRs as functions of urine creatinine and protein concentrations.

**Table 1:** Demographic characteristics and clinical information for the study population (n = 93).

Maternal demographics	
Age (years)	30.8 (19–44)
Gravidity	2 (1–3)
Parity	0 (0–1)
Gestational age (weeks)	40.0 (37.0–42.0)
Body mass index (kg/m^2^)	30.9 (21.3–54.0)
Race/Ethnicity	
• White	69 (74.2%)
• Hispanic	18 (19.4%)
• African American	2 (2.2%)
• Other	4 (4.3%)

Data are presented as n (%), mean (range), or median (IQR).

### Elevated uPCR ≥ 0.3 was Commonly Observed among Healthy Women with Uncomplicated Term Pregnancies

Two-thirds (63/93) of women had a least one uPCR ≥ 0.3 during hospitalization. One-third (31/93) of women had an elevated uPCR ≥ 0.3 at admission, including 39.1% (9/23) of women admitted for scheduled cesarean delivery who were not in labor. 57.0% (53/93) had a uPCR < 0.3 on admission, but thereafter had at least one elevated uPCR during the hospitalization. 23.7% (22/93) had an elevated uPCR in the recovery or postpartum period only. Only 32.3% (30/93) of these healthy women with low-risk, uncomplicated pregnancies had all measured uPCR values < 0.3 throughout their hospital course. There were no significant differences in demographic features or clinical outcomes between women who had any uPCR ≥ 0.3 at some point during their hospital course versus those who did not (Table 2).

**Table 2:** Comparison of women with or without elevated urinary protein/creatinine ratio (uPCR).

	uPCR ≥ 0.3 (n = 63)	uPCR < 0.3 (n = 30)	p-value
**Age (years)**	31.5 (30.8–32.2)	29.2 (28.2–30.2)	0.06^a^
**Gravidity**	2 (1–3)	2 (1–3)	0.75^b^
**Parity**	0 (0–1)	0 (0–1)	0.77^b^
**Body mass index (kg/m**^**2**^**)**	31.0 (30.4–31.6)	32.3 (31.0–33.6)	0.36^a^
**White race**	47 (74.6%)	22 (73.3%)	0.11^c^
**Experienced labor**	47 (74.6%)	23 (73.3%)	1.00^c^
**Vaginal delivery**	33 (52.4%)	20 (66.7%)	0.26^c^
**Heart rate (beats/min)**	86.4 (88.5–84.3)	82.1 (79.4–84.5)	0.18^a^
**Temperature (°F)**	98.1 (98.0–98.2)	98.3 (98.2–98.4)	0.06^a^
**Respiratory rate (breaths/min)**	19.0 (18.8–19.2)	18.7 (18.4–20.0)	0.31^a^
**Systolic blood pressure (mmHg)**	122.7 (124.7–120.7)	121.5 (119.7–123.3)	0.65^a^
**Diastolic blood pressure (mmHg)**	73.3 (72.1–74.5)	71.5 (69.5–73.5)	0.44^a^
**Infant birth weight (g)**	3,532 (3,476–3,586)	3,428 (3,358–3,498)	0.12^a^

Data are presented as n (%), mean (±SEM), or median (IQR). Maternal vital signs were collected at the time of hospital admission.

^a^ t test.

^b^ Kruskal-Wallis test.

^c^ Fisher’s exact test.

### Proteinuria Increased during Labor and Further Increased after Delivery

**Fig 2:**
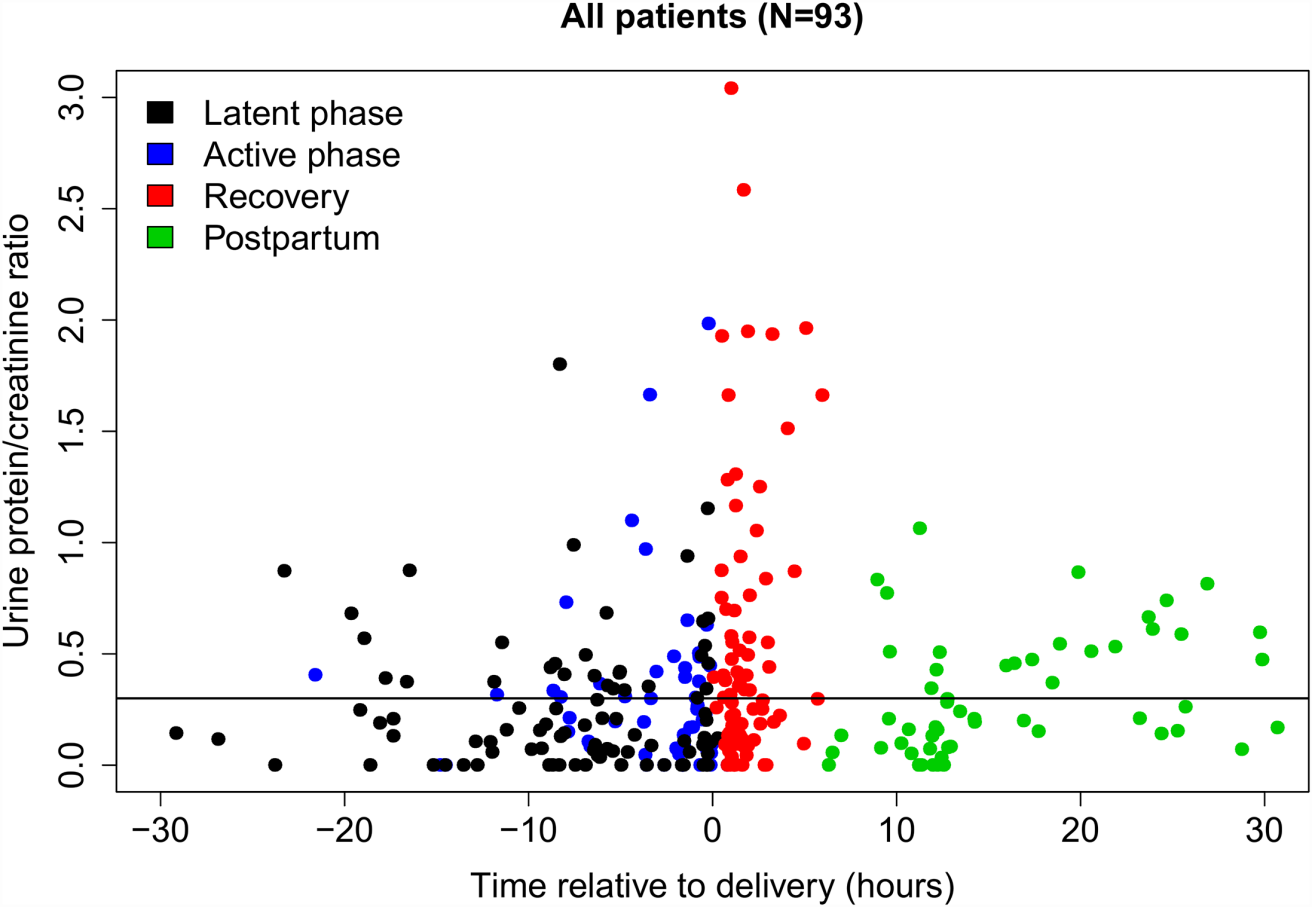
Urine protein/creatinine ratio (uPCR) measurements in all 284 urine samples from 93 women plotted against the timing of the sample collection relative to delivery time. The phase of labor at the time of sample collection is shown for each individual sample by one of four different colors. The horizontal line represents the threshold uPCR value of 0.3.

**Fig 3:**
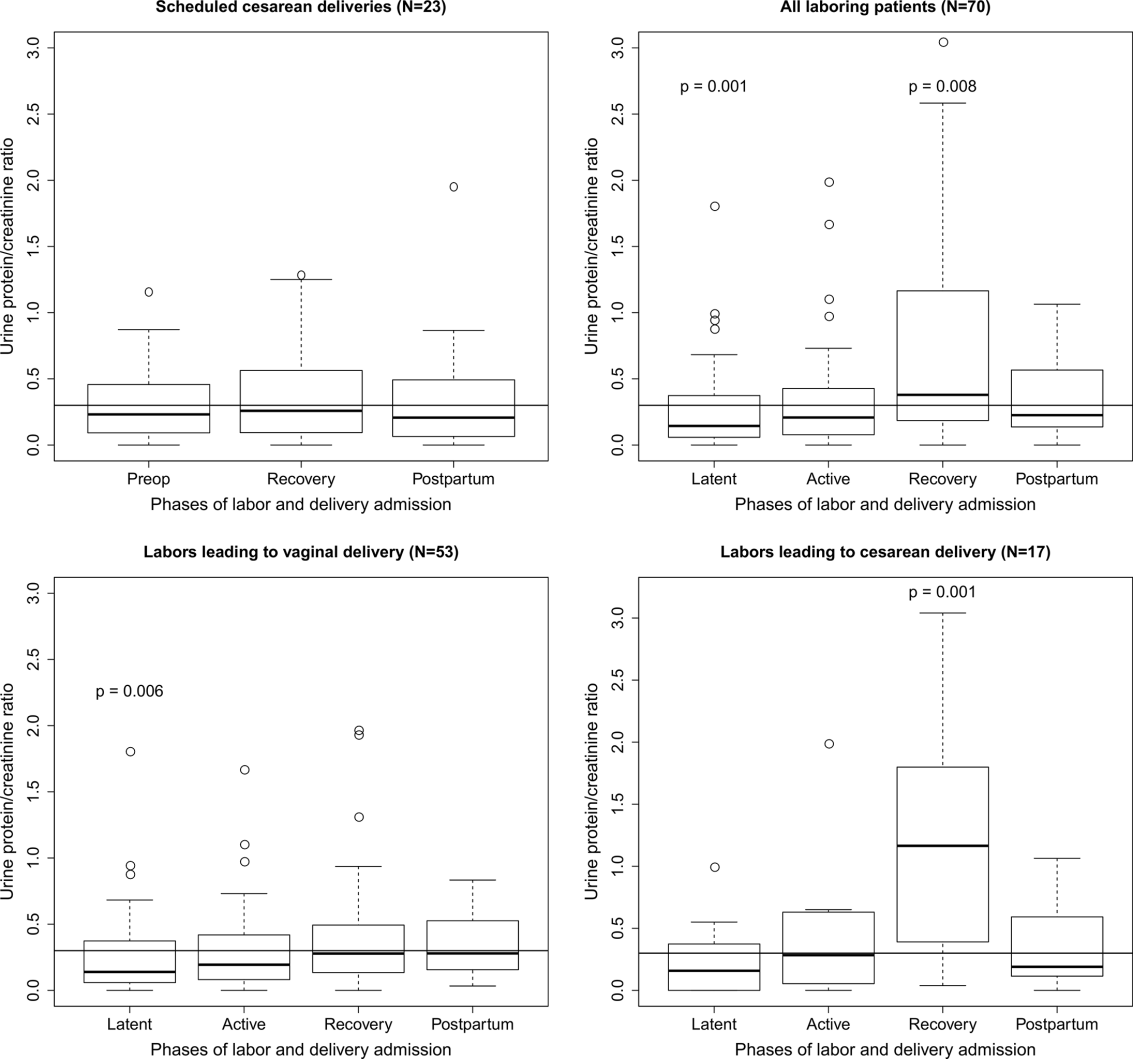
Box plots of urine protein/creatinine ratio (uPCR) measurements by phase of labor at the time of collection and subsequent delivery outcome. uPCR measurements from samples collected before and after delivery among women who had a scheduled cesarean delivery without labor (*top left*), all laboring women (*top right*), laboring women who delivered vaginally (*bottom left*), and laboring women who subsequently delivered by cesarean (*bottom right*) are shown. The horizontal line on each graph represents the threshold uPCR value of 0.3. When the distribution of observed uPCR values is significantly different than the threshold value of 0.3, the p-value (Mann-Whitney u test) is shown.

Fig 2 shows the distribution of uPCR in all 284 samples relative to the delivery time. Although many uPCR measurements were ≥ 0.3 during each phase of labor and during the postpartum period, elevated uPCR was most common during the first 6 h after delivery (designated the recovery period). Among women who donated both an admission and recovery period sample, recovery period uPCRs were significantly greater than admission uPCRs: 0.34 (0.13–0.87) versus 0.15 (0.06–0.39); median (IQR) from n = 67 paired-samples; p = 0.00004, Wilcoxon signed-rank test. From analyses of multiple-imputed datasets, median (IQR) uPCRs increased significantly during active labor and further significantly increased after delivery: latent phase/no labor, 0.15 (0.06–0.32); active phase, 0.29 (0.10–0.58); early postpartum, 0.45 (0.18–1.36); median (IQR) from n = 92 paired-samples, all comparisons significant with p-values from analyses of all multiple-imputed datasets ranging from p = 7.2 x 10−9–0.035 by Wilcoxon signed-rank test. uPCR measurements increased during labor and peaked during the recovery period among laboring women, but not among non-laboring women (Fig 3). The greatest increase in uPCRs was observed among women who labored before delivering by cesarean (Fig 3).

### uPCRs were Not Impacted by Method of Sample Collection and Not Well Predicted by Urine Dipstick

**Fig 4:**
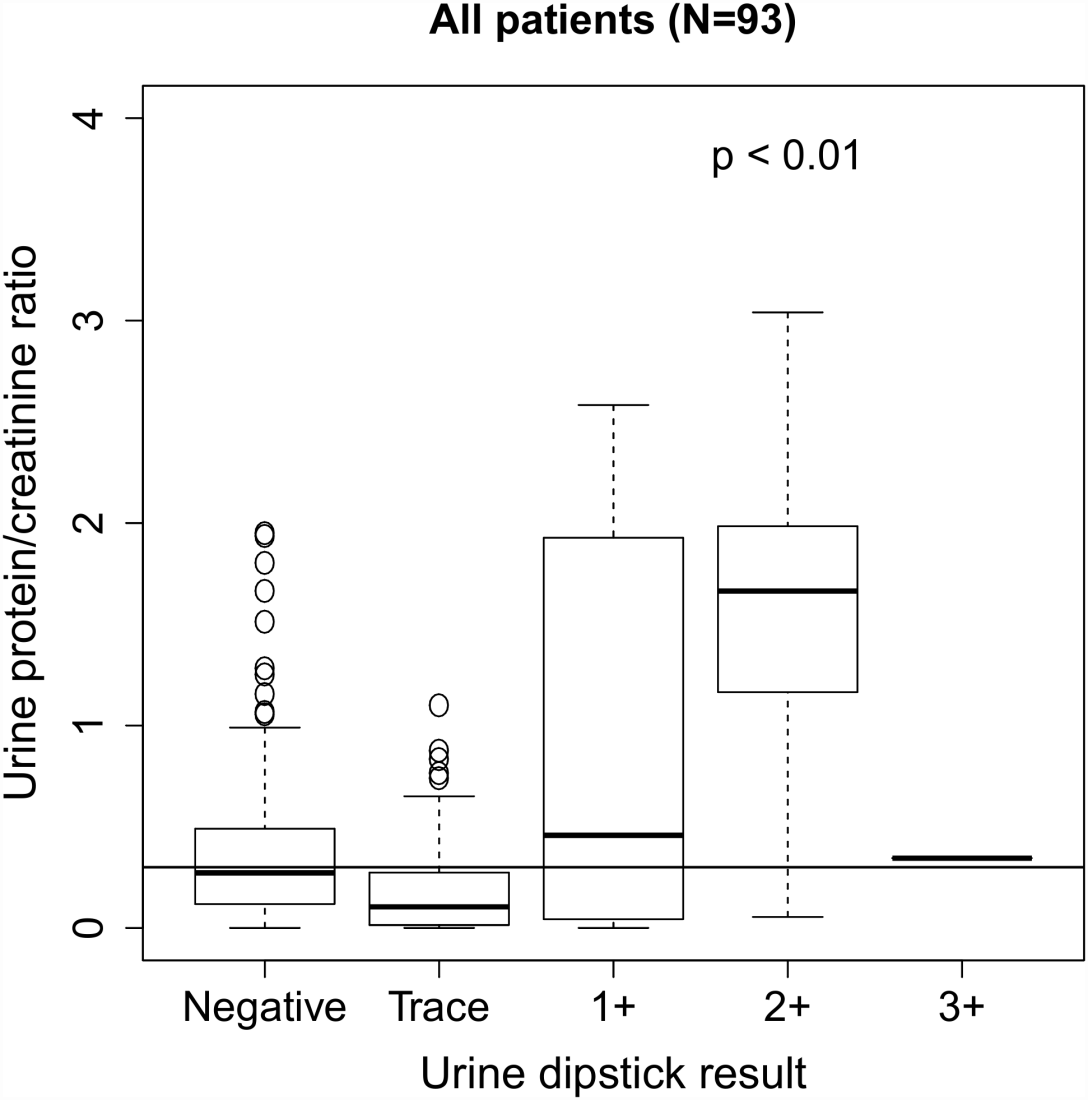
Box plots of urine protein/creatinine ratio (uPCR) measurements by urine protein dipstick result. The horizontal line on each graph represents the threshold uPCR value of 0.3. When the distribution of observed uPCR values is significantly different than the threshold value of 0.3, the p-value (Mann-Whitney u test) is shown. Three uPCR outlier values (4.9, 6.1, and 8.9 in the Negative, 1+, and 2+ groups, respectively) are not shown.

Most (84.1%, 53/63) samples with an elevated uPCR were collected by catheterization. There was no significant difference in uPCR measurements by method of sample collection: 0.22 (0.09–0.48) in samples collected by catheterization (n = 218) versus 0.23 (0.07–0.50) in samples collected by mid-stream clean catch (n = 64); median (IQR); p = 0.831, Mann-Whitney u test. Correlations between uPCR and urine dipstick results are shown in Fig 4 and Table 3. 47.2% (85/180) of samples with a negative dipstick for protein and 21.5% (17/79) of samples with trace proteinuria on dipstick had a measured uPCR ≥ 0.3.

**Table 3:** Urinary protein/creatinine ratio (uPCR) by urine dipstick protein result.

	Proteinuria on dipstick					p-value^a^
	Negative (n = 180)	Trace (n = 79)	1+ (n = 9)	2+ (n = 9)	3+ (n = 1)	
**uPCR**	0.27 (0.12–0.49)	0.10 (0.01–0.27)	0.46 (0.04–1.93)	1.70 (1.17–1.98)^b^	0.30 (N/A)	< 0.0001
**uPCR ≥ 0.3**	85/180 (47.2%)	17/79 (21.5%)	6/9 (66.7%)	7/9 (77.8%)	1/1 (100%)	

Data are median (IQR) or n (%). N/A: not applicable.

^a^ Kruskal-Wallis test.

^b^ Significantly greater than uPCR = 0.3 (p < 0.01, Mann-Whitney u test).

## Discussion

uPCR measurements ≥ 0.3 were common among healthy women with low-risk, uncomplicated pregnancies at term. Women experiencing labor and delivery more often had elevated uPCR measurements. uPCRs were highest within 6 hours of delivery after labor. Consistent with previous reports [13], uPCR measurements were similar regardless of the method of urine sample collection (catheterization or clean catch) among women with intact membranes and no visible vaginal bleeding. A negative or trace urine dipstick result was not reliable to exclude uPCR ≥ 0.3.

Strengths of this study include the prospective study design allowing repeated measurements of uPCRs over time to reveal changes in proteinuria during labor and delivery. The study size (n = 93 with 284 samples collected) is similar to or larger than other relevant studies of uPCR [4,6,13]. Finally, the focus on healthy women with low-risk, uncomplicated pregnancies at term allows characterization of normal physiology as relates to proteinuria during labor and delivery. Our findings should be generalizable to other low-risk populations.

Limitations of this study include that many women declined sample collection by catheterization in the postpartum period and, as such, data analysis of later postpartum specimens (> 6 hours after delivery) is limited. Additionally, a single assay method was used for measurement of uPCRs. This method has demonstrated falsely elevated results in dilute samples [6]. Finally, the study was not designed to identify the cause of proteinuria. We also did not systematically measure hematuria in these specimens. Catheterization for specimen collection of most specimens in the study ensured that if blood was present in a specimen it likely represented hematuria rather than contamination from uterine/vaginal bleeding. Transient hematuria among laboring women may be due to renal hemoglobin excretion that occurs during exhaustive exercise [14].

The mechanism by which proteinuria is increased in laboring women is unknown, but may be related to an increase in glomerular protein filtration and/or a decrease in renal tubular protein reabsorption in labor as has been suggested for exercise-induced functional proteinuria [14]. Our study is the second to report that 39% of nonlaboring women at term had an elevated uPCR > 0.3 [6]. We add to this finding by demonstrating that uPCRs are increased during labor and the early postpartum period among healthy women with normal pregnancies. These findings are important given the context that pregnant women after 37 weeks gestational age are routinely evaluated for new onset hypertension, diagnosed with preeclampsia based upon a single uPCR ≥ 0.3, and then delivered consistent with existing guidelines. Our study shows that a single uPCR ≥ 0.3 occurs commonly in uncomplicated pregnancies before, during, and after labor, which decreases the utility of the now guideline-supported uPCR with an arbitrary yet convenient threshold criterion value of 0.3. As previously reviewed, proteinuria may increase due to several etiologies without signifying pathology due to increased basement membrane permeability at the glomeruli, increased flow through the glomeruli due to increased cardiac output (for instance, during the second stage of labor which may be similar to the work of exercise) or increased emotional stress associated with increased circulating catecholamines [15]. Our study was not designed to determine the etiology of the observed proteinuria, but was intended to determine, as we did, that elevated uPCR is common in late pregnancy and during the peripartum period. Performance of this study in healthy women with uncomplicated pregnancies demonstrates that this proteinuria often may not indicate underlying pathology. Further studies may call into question the entire role of proteinuria measurement as a criterion for the diagnosis of preeclampsia, especially in late pregnancy. It would also be valuable to identify the protein components of urine that may better specify the underlying etiology of observed proteinuria and the likelihood of any observed proteinuria representing meaningful pathology rather than normal physiology.

Consistent with prior observations [6], samples that were dilute as defined by low creatinine concentrations were more likely to have uPCR levels suggestive of significant proteinuria. In our cohort, the value of urine creatinine at which 95% of calculated uPCRs were ≥ 0.3 was 30 mg/dL. The precise cause of this phenomenon is unclear, but it may be related to conditions of low ionic strength leading to an enhanced interaction between proteins and the pyrocatechol violet dye [10]. Our results draw further attention to the point that practicing obstetricians using the uPCR should know the assay used in their laboratory and the assay limitations, as was also previously suggested [15]. Based on our study and other data [6], we suggest that specimens with a creatinine concentration ≤ 30 mg/dL be considered inadequate for the accurate determination of uPCR using the pyrocatechol violet-dye method for protein estimation. As was previously suggested [15], obstetricians may want to determine the 95% threshold of their laboratory proteinuria assay and make note that a dual cut-off for uPCR is required, with a low cut-off value needed to confidently exclude pathologic proteinuria and higher cut-off value needed to confirm the presence of likely pathologic proteinuria [16]. The exact cut-off values may need to be determined for each laboratory’s chosen uPCR assay by correlation to proteinuria values from concurrently obtained 24 hour urine collections. This determination for uPCR cut-off values will likely remain complicated by the fact that proteinuria varies with the time of day, with activity, with gestational age, and now we also show with labor and delivery [15,17].

An elevated uPCR (defined as ≥ 0.3) is common among healthy women with uncomplicated pregnancies at term, and is seen even more commonly during and immediately after labor. Moreover, some protein assay methods may perform poorly when the urine sample is excessively dilute, resulting in false-positive uPCR results. Taken together, these data suggest that the uPCR threshold of ≥ 0.3 is an unreliable measure of proteinuria at term, and that obstetric care providers should exercise caution when using uPCR as a diagnostic criterion for preeclampsia at term or during the peripartum period. It is also interesting to consider that the standard threshold value of 300 mg/24 h for abnormal proteinuria was established using urine specimens collected before the onset of labor and may be too low during the peripartum period. Further studies are needed to characterize to what degree proteinuria may be physiologic in both high- and low-risk populations at term and during the peripartum period.

## Supporting Information

S1 File STROBE checklist. (DOC)

S2 File Study dataset. (XLSX)
